# N-terminal fragment of B-type natriuretic peptide (NT-proBNP), a marker of cardiac safety during antipsychotic treatment

**DOI:** 10.1186/1744-859X-4-10

**Published:** 2005-05-09

**Authors:** Stefan Kropp, Argyro Tountopoulou, Udo Schneider, Ralf Lichtinghagen

**Affiliations:** 1 Department of Clinical Psychiatry and Psychotherapy, Hannover Medical School, 30623 Hannover, Germany; 2 Department of Psychiatry and Psychotherapy, Lübbecke Medical Hospital, Virchowstr. 65, 32312 Lübbecke, Germany; 3 Department of Clinical Chemistry, Hannover Medical School, 30623 Hannover, Germany

## Abstract

**Background:**

The potential cardiotoxicity of antipsychotic drugs is well known. The N-terminal fragment of B-type natriuretic peptide (NT-proBNP) is considered to be a possible biomarker in clinical practice for the diagnosis and prognosis in patients with suspected heart failure. This pilot evaluation tests the influence of antipsychotic drugs on NT-proBNP concentration in view of the hypothesis that NT-proBNP could be used as marker for the tolerability and safety of antipsychotic medications.

**Methods:**

On a routine basis, patient's blood samples were examined for NT-proBNP on days 0, 7 and 21 after initiation of a new antipsychotic monotherapy. All plasma samples were analysed for NT-proBNP using an electrochemiluminiscence immunoassay "ECLIA" (proBNP kit, Roche Diagnostics, Mannheim, Germany) on an Elecsys 2010 analyser.

**Results:**

A difference was found in NT-proBNP values at day 0 between patients younger versus older than 40 years. Also women had comparatively lower NTproBNP on days 7 and 21. Smokers' levels of NT-proBNP values decreased more from day 0 to day 7.

**Conclusion:**

Our results suggest that antipsychotic medication influences the plasma concentration of NT-proBNP, suggesting a possible method to identify high-risk-patients for cardiovascular adverse effects due to antipsychotic medication. Larger studies should further test this hypothesis.

## Background

The potential cardiotoxicity of antipsychotic drugs has been recognized since the 1960s [1]. The most known of these cardiological side effects is the QT-prolongation in the electrocardiogram (ECG), which predisposes to a life-threatening ventricular arrhythmia known as Torsades de Pointes (TdP) and sudden death. Other cardiac adverse effects related to antipsychotic medication such as myocarditis and cardiomyopathy with sometimes fatal effect have been recently reported [2]. Brain or B-type natriuretic peptide (BNP) belongs to a family of vasoactive peptides and is primarily synthesized by the ventricular myocardium [3]. It acts as a key regulator in the homeostasis of water and salt excretion and in the maintenance of blood pressure [4] mainly by inhibiting the renin-angiotensin-aldosteron-axis and blocking the cardiac sympathetic nervous activity [4,5]. Its synthesis and secretion as proBNP is activated by myocyte stretch [6]. In this process it is split into physiologically active BNP and the N-terminal fragment NT-proBNP. Both are considered to be valuable biomarkers in clinical practice for the prediction of disease state and prognosis in patients with suspected heart failure [5]. Although adequate comparisons standing shoulder to shoulder have not been done [5], amino-terminal pro-brain natriuretic peptide (NT-pro-BNP) seems to provide very similar information to BNP. It is therefore a promising alternative marker for the detection of left-ventricular dysfunction [7]. According to other authors [8], the proportional and absolute rise of NT-proBNP values above normal plasma levels in cardiac impairment (including NYHA Class I) exceeds the rise of BNP levels. This suggests that NT-proBNP may be a more accurate marker of early cardiac dysfunction than BNP.

The aim of this clinical evaluation was to test the influence of antipsychotic drugs on NT-proBNP concentration with the hypothesis that NT-proBNP could be used as marker for tolerability and safety of antipsychotic medication.

## Methods

Blood samples of 36 patients, who were treated with first (FGAs) or second-generation antipsychotics (SGAs), were selected on a routine basis.

### Inclusion Criteria

Patients had the diagnosis of schizophrenia, schizoaffective or affective disorder according to ICD-10 with the need of an antipsychotic treatment. Their age ranged from 18 to 66 years. Patients with a previous history of major head injuries or neurological disorders, diabetes, current or previous substance misuse and patients receiving a combination of antipsychotics were excluded from this analysis. There was no washout period in patients who were treated with other antipsychotics before.

### Blood samples examination

The blood samples had to be examined for NT-proBNP during the routine laboratory tests in a three-week-pattern. Day 0 was the day of the new treatment with an antipsychotic, whether a first or a second-generation antipsychotic drug. Blood samples were scheduled for day 0, 7 and 21 for each individual patient.

### Analysation technique

Venous blood was drawn in the early morning after an overnight fast and centrifuged at 2000 g for 15 minutes to remove RBCs; the obtained clear plasma fraction was stored at -20° until the time of assay. All plasma samples were analysed for NT-proBNP using an electrochemiluminiscence immunoassay "ECLIA" (proBNP kit, Roche Diagnostics, Mannheim, Germany) on an Elecsys 2010 analyser. The assay had a measuring range from 0.6 to 4130 pg/ml and a functional sensitivity of <50 pg/ml. All assays were performed blind to clinical information on the patients.

### Statistical Analysis

Data were analysed using nonparametric statistics, because data were only partly normally distributed. Group comparisons were examined using the Mann-Whitney test (two-tailed) for unpaired and Wilcoxon test for paired groups. A Bonferonni correction was taken into account in case of multiple tests. We performed 39 tests in total. After a Bonferonni correction all statistical tests were considered significant at the 0,0013 (0,05/39) probability level. The SPSS 10.0 package was used throughout.

## Results

### Patients and treatment

The mean age of the patients was 42,3+/-15,6 years (range:19–74). There were 16 men (44,4%) and 20 women (55,6%). Seventeen patients were smokers (47,2%) and 7 (19,4%) had cardiovascular disease (5: hypertension, 1: heart failure, 1: pacemaker). The administered medication was as follows: 8 patients received FGAs (haloperidol: 3, flupentixol: 4), 5 patients amisulpride, 11 patients risperidone, 4 patients clozapine, 6 patients olanzapine and 2 patients received quetiapine.

### NT-proBNP measurement

The median measured NT-proBNP value for the group of FGAs was 28,00 at day 0 (mean = 55,63 +/-67,45, range: 5–165), 13,50 at day 7 (mean = 18,13 +/-15,70, range 5–54) and 24,00 at day 21 (mean = 59,00 +/-75,92, range: 5–188). The median measured NT-proBNP value for the group of amisulpiride and risperidone was 20,50 at day 0 (mean = 31,75 +/-35,64, range: 9–151), 15,00 at day 7 (mean = 26,94 +/-40,73, range: 5–176) and 23,50 at day 21 (mean = 24,31 +/-18,94, range: 5–75). The median measured NT-proBNP value for the group of olanzapine, clozapine and quetiapine was 25,00 at day 0 (mean = 27,33 +/-17,72, range: 6–59), 28,50 at day 7 (mean = 31,25 +/-20,24, range: 6–69) and 25,50 at day 21 (mean = 55,92 +/-92,13, range: 5–335). The performance of the Mann and Whitney test showed no statistical differences in NT-proBNP values of each day between the different groups of antipsychotics.

### The impact of age

The median NT-proBNP value of patients younger than 40 years was 13,50 on day 0 (mean = 17,25 +/-10,27, range: 6–40), 14,50 at day 7 (mean = 19,83 +/-17,17, range: 6–69), and 23,50 on day 21 (mean = 35,33 +/-49,44, range: 6–188). For patients older than 40 years the median NT-proBNP value was 28,50 on day 0 (mean = 44,75 +/-47,28, range: 5–165), 21,50 on day 7 (mean = 29,71 +/-34,85, range: 5–176) and 26,50 on day 21 (mean = 46,17 +/-72,50, range: 5–335).

Comparing the median NT-proBNP values between the two age groups (Mann and Whitney test) on day 0 (13,50 vs. 28,50, p = 0,032), on day 7 (14,50 vs. 21,50, p = 0,311) and on day 21 (23,50 vs. 26,50, p = 0,987), no significant differences were found (after Bonferonni correction).

In the subgroup of patients younger than 40 years old, the NT-proBNP values showed a trend to increase but the performance of the Wilcoxon test demonstrated no significant differences of the NT-proBNP values between days 0 and 7 (13,50 vs. 14,50, p = 0.894), 7 and 21 (14,50 vs. 23,50, p = 0.119) and 0 and 21 (13,50 vs. 23,50, p = 0.197).

In the subgroup of older patients there were also no significant differences (after Bonferonni correction) of NT-proBNP values between day 0 and day 7 (28, 50 vs. 21,50, p = 0,042), from day 7 to 21 (21,50 vs. 26,50, p = 0.417) and between day 0 and 21 (28,50 vs. 26,50, p = 0.533).

### The impact of sex

The median NT-proBNP value of men was 14,00 on day 0 (mean = 19,63 +/-14,79, range: 5–59), 18,00 on day 7 (mean = 23,38 +/-19,48, range: 5–69), and 18,50 on day 21 (mean = 52,56 +/-89,14, range: 5–335). The median NT-proBNP value of women was 28,00 on day 0 (mean = 48,35 +/-50,26, range: 9–165), 20,00 on day 7 (mean = 28,85+/- 36,99, range: 5–176), and 27,00 on day 21 (mean = 34,55+/-37,46, range: 5–172).

Comparing the median NT-proBNP values between men and women (Mann and Whitney test) on day 0 (14,00 vs. 28,00, p = 0,018), on day 7 (18,00 vs. 20,00, p = 0,789) and on day 21 (18,50 vs. 27,00, p = 0,459), no significant differences were found (after Bonferonni correction).

The NT-proBNP value in men showed a trend to increase over time, but the performance of the Wilcoxon test revealed no significant differences of NT-proBNP value from day 0 to 7 (14,00 vs. 18,00, p = 0,506), from day 7 to 21 (18,00 vs. 18,50, p = 0,348) and from day 0 to 21 (14,00 vs. 18,50, p = 0,300).

Women showed a decrease in NT-proBNP value between day 0 and 7 (28,00 vs. 20,00, p = 0,017) but the difference was not significant (after Bonferonni correction). In the same way no significant differences were revealed in NT-proBNP values from day 7 to 21 (20,00 vs. 27,00, p = 0.396) and from day 0 to 21 (28,00 vs. 27,00, p = 0.422).

### Smoking and NT-proBNP

The median NT-proBNP value of non-smokers was 30,00 at day 0 (mean = 41,05+/-44,58, range: 9–165), 26,00 at day 7 (mean = 35,74+/- 39,05, range: 6–176), and 26,00 at day 21 (mean = 53,74+/-79,46, range: 5–335). The median NT-proBNP value of smokers was 21,00 at day 0 (mean = 29,47+/-36,79, range: 5–161), 15,00 at day 7 (mean = 16,00 +/- 7,97, range: 5–31), and 16,00 at day 21 (mean = 30,06+/-43,35, range: 5–188).

Comparing the median NT-proBNP values between smoking and non-smoking patients (Mann and Whitney test) at day 0 (21,00 vs. 30,00, p = 0,271), at day 7 (15,00 vs. 26,00, p = 0,045) and at day 21 (16,00 vs. 26,00, p = 0,285) no significant differences were found (after Bonferonni correction).

The decrease of the NT-proBNP value from day 0 to 7 in smoking patients was greater (from 21,00 to 15,00, p = 0.038) than in non-smoking patients (from 30,00 to 26,00, p = 0.647) but this decrease was in neither group significant (after Bonferonni correction).

### History of cardiovascular disease and NT-proBNP

The median NT-proBNP value of patients with a positive cardiovascular history (hypertension, heart failure, arrhythmias) was 67,00 on day 0 (mean = 90,57+/-66,48, range: 9–165), 39,00 on day 7 (mean = 56,29+/-56,91, range: 9–176) and 27,00 on day 21 (mean = 37,71+/-36,13, range: 5–101). The median NT-proBNP value of patients with a negative cardiovascular history was 20,00 on day 0 (mean = 22,31+/-14,42, range: 5–59), 16,00 on day 7 (mean = 19,21+/-13,05, range: 5–69) and 25,00 on day 21 (mean = 43,72+/-70,89, range: 5–335).

Patients with a positive cardiovascular history had higher NT-proBNP values in comparison to patients without cardiovascular diseases history on day 0 (67,00 vs. 20, 00, p = 0,005) at day 7 (39,00 vs. 16, 00, p = 0,026) and at day 21 (27,00 vs. 25,00, p = 0,725). The mean age was 60,7+/-8,4 and 37,7+/-13,5 years for the subgroup with positive and negative cardiovascular history respectively, which was significant different (p = 0,000). There were neither differences between the sexes (p = 0,433) nor differences in therapy (p = 0.387), nor in smoking habits (p = 0.105) between the two groups. There were no statistical differences in NT-proBNP values over the 3 weeks in neither of the two groups. In patients with a negative cardiovascular history the median NT-proBNP value on day 21 was greater than the one in the respective group at baseline measurement.

## Discussion

The aim of this pilot study testing the use of NT-proBNP in clinical routine was to investigate whether antipsychotics influence NT-proBNP concentrations. This might lead to the use of NT-proBNP as a marker for the detection of high-risk patients regarding cardiovascular adverse effects in patients receiving antipsychotic drugs. No statistical differences in the NT-proBNP values were found among the different groups of antipsychotics. Patients older than 40 years had higher values in comparison to younger patients (mean = 44,75+/-47,28 vs.17,25+/-10,27, p = 0,032 at day 0, mean = 29,71+/-34,85 vs. 19,83+/-17,17 p = 0,311, on day 7, mean = 46,17+/-72,50 vs. 35,33+/-49,44, p = 0,987 at day 21). In younger patients NT-proBNP values showed a trend to increase over time. Women had higher values in comparison to men (mean = 48,35+/-50,26 vs. 19,63+/-14,79, p = 0,018 at day 0, mean = 28,85+/-36,99 vs. 23,38+/-19,48, p = 0,789 at day 7, mean = 34,55+/-37,46 vs. 52,56+/-89,14, p = 0,459 on day 21). NT-proBNP values in men showed a trend to increase over time. Non-smoking patients had higher values in comparison to smoking ones (mean = 41,05+/-44,58 vs. 29,47+/-36,79, p = 0,271 at day 0, mean = 35,74+/-39,05 vs. 16,00+/-7,97, p = 0,045 at day 7, mean = 53,74+/-79,46 vs. 30,06+/-43,35, p = 0,285 at day 21). Smoking patients showed a greater decrease of the NT-proBNP values from day 0 to day 7(mean = 29,47+/-36,79 at day 0 to mean = 16,00+/-7,97 at day 7, p = 0,038) in comparison to non-smoking ones (mean = 41,05+/-44,58 at day 0 to mean = 35,74+/-39,05 at day 7, p = 0,647). Patients with a positive cardiovascular history had higher values in comparison to patients with a negative one (mean = 90,57+/-66,48 vs. 22,31+/-14,42, p = 0,005 on day 0, mean = 56,29+/-56,92 vs. 19,21+/-13,05, p = 0,026 at day 7, mean = 37,71+/-36,13 vs. 43,72+/-70,89, p = 0,725 at day 21). These differences were reduced over time.

BNP and NT-proBNP are new cardiac markers with a number of potential applications in both the clinical diagnosis and prognostic assessment of heart failure. In early pilot studies raised concentrations of BNP (with a sensitivity of 97% and a specificity of 84% (9)) distinguished heart failure from other causes of dyspnoea more accurately than left-ventricular ejection fraction, atrial natriuretic peptide (ANP) and N-terminal ANP did. In comparison with history, clinical signs and tests a high BNP concentration was the strongest predictor of underlying heart failure [10]. In patients with dyspnoea on exercise NT-proBNP measurement showed a sensitivity of 75% with a specificity of 79% and a negative predictive value of 99% for the detection of high-grade left-ventricular pump-dysfunction [11]. Because of the high negative predictive value of the marker a high-grade left-ventricular dysfunction could be safely ruled out in symptomatic patients with normal concentrations of NT-proBNP [10]. Elevated NT-proBNP concentration has been proven to be a good prognostic marker after acute coronary syndromes or myocardial infarction as well as a marker for patients with chronic heart failure and decreased left-ventricular dysfunction [10]. This fact could facilitate the identification of patients at risk and improved care during follow-up of these patients. Interestingly a recent study has shown that not only the initial concentrations but also the follow-up measurements compared with the initial ones are of prognostic importance [10]. Furthermore Throughton et al. [12] have shown that in patients with impaired left-ventricular systolic function and established symptomatic heart failure drug treatment guided by plasma NT-proBNP concentrations reduced the total number of cardiovascular events more than a treatment guided by clinical judgment did.

Minor cardiovascular adverse effects from antipsychotic drugs are common. They include postural hypotension and tachycardia due to anticholinergic or alpha1-adrenoreceptor blockade. They may occur in the majority of patients at therapeutic dosages [13]. Among several ECG abnormalities induced by antipsychotic drugs (AV-Blocks, widening of QRS-Complexes) the QT interval prolongation is the most vital. Most of antipsychotic drugs have been associated with QT prolongation, sometimes in a dose-dependent fashion, and some have been linked (with varying levels of confidence) to TdP and sudden death [14]. At the same time not only antipsychotics but also other psychotropic drugs such as tricyclic and tetracyclic antidepressants can cause prolongation of the QT interval [15]. The QT interval on the ECG is the time from the onset of ventricular depolarization to completion of reporalization. The prolongation of the QT interval is associated with an increased risk of dysrhythmias, especially to mention TdP, and of sudden cardiac death. The risk of electrical heart instability can be increased in pathological myocardial tissue, as for example in myocardial hypertrophy and ischaemia and in coronary atherosclerosis. This is because of the loss of membrane integrity, which disrupts both depolarization and repolarization [16]. Heart muscle disorders such as myocarditis and cardiomyopathy have been recently reported as adverse effects of clozapine, but also of other antipsychotic drugs (i.e. risperidone, haloperidol, olanzapine, quetiapine), although these associations were much weaker than for clozapine [2]. These adverse effects, which potentially lead to a heart failure, could add to the already increased cardiovascular risk of schizophrenic patients [16], resulting in lethal effects.

Our results suggest that antipsychotic medication influences the plasma concentrations of NT-proBNP. NT-proBNP concentrations are normally higher in women and in older people [17]. This impact of age and sex on NT-proBNP plasma levels, though not significant, can be seen in the baseline measurements of our patients. Older patients had higher NT-proBNP values on day 0 in comparison to younger patients(mean = 44,75+/-47,28 vs. 17,25+/-10,27, p = 0,032). Women had higher NT-proBNP values on day 0 in comparison to men (mean = 48,35+/-50,26 vs. 19,63+/-14,79, p = 0,018). The trend of an increase of the NT-proBNP values over time in male and younger patients diminished these differences in follow up measurements one and three weeks after administering the antipsychotic medication. That could not be expected, because antipsychotics differ importantly in pharmacology and widely in chemical structure. Because of that, it is unlikely that all of them have the same effects on heart function and accordingly on NT-proBNP concentrations in all patients. Among the different groups of antipsychotics patients of the group who received clozapine showed a remarkable increase of NT-proBNP plasma levels on day 7 in contrast to the decreasing values in the other groups of antipsychotics by comparable values on day 0. This is consistent with literature about an association of clozapine with cardiomyopathy and myocarditis to a severe [18] and a greater degree as other antipsychotics [2]. Smoking patients had higher values in comparison to non-smoking ones. The decrease of NT-proBNP plasma levels in smoking patients one week after receiving antipsychotic medication was greater than the respective one of non-smoking patients (from 29,47+/-36,79 to 16,00+/-7,97, p = 0,038 vs. from 41,05+/-44,58 to 35,74+/-39,05, p = 0,647). Nicotine induces the liver enzyme system (CYP1A2), which is used for the metabolisation of several antipsychotics, resulting in lower plasma levels of these drugs. The lower plasma levels of the quickly degraded antipsychotics could cause the lower NT-proBNP plasma levels in smokers.

Our paper has certain limitations. The number of patients was small. There was no washout period for patients taken other antipsychotic drugs before the start of the evaluation and patients with other than antipsychotic co-medication or other medical illnesses, which might influence the NT-proBNP levels, were not excluded. NT-proBNP is proved to be stable in EDTA plasma for a period between 6 and 24 hours [4] or even for 3 days at room temperature or longer at 4°C [19]; whether the stability of NT-proBNP decreases when stored at -20°C for a longer time is not known.

## Conclusion

Despite the limitations of this study and the non-significant results in this small sample the measurement of the NT-proBNP concentration at baseline and after the beginning of antipsychotic medication seems to be a promising method to identify patients with an increased risk of dangerous cardiovascular adverse effects due to antipsychotic medication. Studies with larger number of patients, which would also examine the clinical impact of the NT-proBNP balances on the heart dysfunction in patients treated with antipsychotics, should test the hypothesis of this evaluation.

## Competing interests

The authors have obtained the NT-proBNP reagent from Roche Diagnostics.

## Authors' contributions

SK conceived and designed the study and helped to draft the manuscript. AT participated in designing the study, performed the statistical analysis and drafted the manuscript. US collected and interpreted the clinical data and revised the manuscript. RL carried out the NT-proBNP tests and corrected the manuscript. All authors read and approved the final manuscript.
